# Comparison between optical coherence tomography angiography and fluorescein angiography findings in retinal vasculitis

**DOI:** 10.1186/s40942-018-0117-z

**Published:** 2018-04-16

**Authors:** Julio Zaki Abucham-Neto, André Augusto Miranda Torricelli, Aline Cristina Fioravanti Lui, Sarah Napoli Guimarães, Heloisa Nascimento, Caio Vinícius Regatieri

**Affiliations:** 1 Departamento de Oftalmologia, Universidade Federal de São Paulo, Rua Botucatu, 821 - Vila Clementino, São Paulo, SP 04023-062 Brazil; 2 Departamento de Oftalmologia, Faculdade de Medicina do ABC, São Paulo, Brazil; 3 Departamento de Oftalmologia, Universidade de São Paulo, São Paulo, Brazil; 4 Departamento de Oftalmologia, Faculdade de Ciências Médicas da Santa Casa de São Paulo, São Paulo, Brazil

**Keywords:** OCT angiography, En Face, Retinal vasculitis, Angiofluoresceinography, Uveitis, Behçet, Takayashi arteritis, Toxoplasmosis, Sarcoidosis

## Abstract

**Background:**

To describe optical coherence tomography angiography (OCT-A) findings in patients with retinal vasculitis and to compare them to current fluorescein angiography (FA) findings.

**Methods:**

This was an observational case series. Nineteen eyes in 10 patients with retinal vasculitis of various etiologies were imaged with FA (TRC-50DX, Topcon) and OCT-A (SD-OCT, Optovue). The images were reviewed and analyzed.

**Results:**

The mean age was 36 years (range 24–67 years); there were three males and seven females. The primary vessels involved were veins (89%). Fourteen eyes (74%) had active inflammatory disease during the study period, with signs of vascular sheathing and perivascular leakage on FA. Interestingly, in this group, OCT-A was not able to detect clear signs of active inflammation around the affected vessels. Nevertheless, OCT-A was able to detect secondary lesions in fourteen eyes (74%), including some findings not clearly shown on FA. Most of these were within the macular area. OCT-A was particularly effective in cases of capillary dropout, increased foveal avascular zone, telangiectasias, shunts, and areas of neovascularization.

**Conclusion:**

FA remains an essential complementary exam for detection of retinal vasculitis. However, OCT-A extends FA findings and affords better assessment of secondary complications.

## Background

Retinal vasculitis is an inflammatory condition involving retinal vessels that potentially leads to blindness [1, 2]. There are several etiologies, including local ocular diseases, systemic autoimmune conditions and infections. The characteristic fundoscopic finding is perivascular sheathing that is best demonstrated by vascular staining and leakage on fluorescein angiography (FA) [3]. Various complications can occur, including macular edema, vascular abnormalities, retinal ischemia, retinal hemorrhages and cotton-wool spots. All these currently require both Optical Coherence Tomography (OCT) and FA as complementary exams to detect primary vascular lesion activity and to assess severity of secondary retinal complications. FA has some well-known limitations, particularly that it is an invasive exam with risks associated with infusion of dye into veins. It is also time-consuming and relatively expensive [4].

OCT-Angiography (OCT-A) is a new noninvasive diagnostic imaging technique that has recently emerged to assist in the evaluation of chorioretinal diseases. This new technology constructs three-dimensional retinal and choroidal capillary images by Doppler OCT in a matter of seconds, detecting erythrocyte movement in retinal blood vessels, without the use of contrast [5].

The diagnosis of vasculitis is often confirmed by findings on FA, such as dye leakage or perivascular staining, therefore, FA remains the diagnostic gold standard [6]. However, secondary complications can be covered by this leakage as well as hemorrhage or media opacities, and the depth of the lesion can be difficult to detect because the segmentation of various layers may not be identified on FA. *En Face* images (OCT scans) can be displaced outward from the internal limiting membrane to the choroid to visualize the vascular plexus and to segment the inner retina, outer retina, choriocapillaris, and other areas of interest [5].

There are few reports of OCT-A findings in patients with retinal vasculitis. Therefore, the objective of this study was to compare OCT-A to the current fluorescein angiography findings in a case series of retinal vasculitis from various etiologies. We aimed to evaluate the accuracy OCT-A for detection of perivascular sheathings and other indirect signs of vascular inflammation and/or secondary complications, including vascular non-perfusion areas, vascular abnormalities and retinal neovascularization.

## Methods

Nineteen eyes in ten patients with retinal vasculitis were imaged with FA and OCT-A at the Uveitis Section at Federal University of São Paulo (UNIFESP). The tests in each patient were all carried out on the same day.

In order to compare the modalities in all cases, we retained all data, even when we encountered difficulties in obtaining good quality images because of media opacity or fixation loss. Complete medical histories and ocular examinations were performed, including best-corrected visual acuity (BCVA), biomicroscopy and fundoscopy.

FA images were obtained with the TRC-50DX (Topcon Medical Systems, Oakland, NJ), after intravenous dye infusion, with photographic documentation of the posterior pole and the peripheral retina, over 10 min. On FA exam, we recorded leakage patterns of retinal vessels, capillary dropout, macular ischemia or edema and other changes related to vasculitis.

OCT-A images were obtained through *En Face* OCT Angiography with SD-OCT XR Avanti (Optovue, Inc, Fremont CA, USA) using the Split Spectrum Amplitude Decorrelation Angiography (SSADA) algorithm to distinguish between static and nonstatic tissue. This algorithm identifies blood flow by calculating the decorrelation of signal amplitude from consecutive B-scans performed in the same retinal acquisition plane [7].

For OCT-A, we analyzed the superficial and deep retinal plexus, choriocapillaris and choroid to identify vascular changes that suggested the presence of local or remote active vasculitis, as well as their secondary complications. Superficial FAZ above 0.27 mm^2^ or deep FAZ above 0.35 mm^2^ were considered increased [8].

A 3 × 3 mm field of view was used for visualization of retinal capillaries in all cases, in order to prioritize the central macular findings. An 8 × 8 mm scan was used to perform large area scans of the retina in those cases that had peripheral macular abnormalities identified on fundoscopy. When extra-macular changes were observed, the same scan was displaced towards that area in order to obtain better records. The same investigator performed all imaging exams, and two experienced investigators analyzed the results.

Approval for this study was obtained from the Ethics Committee at the Federal University of São Paulo (UNIFESP) and the authors followed the tenets of the Declaration of Helsinki.

## Results

Nineteen eyes in ten patients with retinal vasculitis were evaluated. The mean age was 36 years (range: 24–67 years); There were three males and seven females. Six met criteria for Behçet’s disease. The other etiological diagnoses were as follows: one toxoplasmosis, one sarcoidosis, one Takayasu arteritis and one idiopathic (Table 1).

**Table 1:** Demographic and clinical data of patients

	Number of committed eyes	Gender	Vasculitis activity
Behçet disease	12	2 Male 4 Female	7 Active phlebitis 5 Inactive phlebitis
Toxoplasmosis	1	1 Female	1 Active phlebitis
Sarcoidosis	2	1 Female	2 Active phlebitis
Takayasu arteritis	2	1 Female	2 Active arteritis
Undetermined diagnosis	2	1 Male	2 Active phlebitis
Total	19	3 Male 7 Female	17 Phlebitis 2 Arteritis

The primary vessels involved among these nineteen eyes were veins (phlebitis), except for two (10%) in which arteritis was the primary inflammatory sign (bilateral Takayasu arteritis). Fourteen eyes (74%) had active inflammatory disease during the study period, with signs of vascular sheathing and perivascular leakage on FA. Interestingly, in this group, OCT-A did not detect any primary sign of active inflammation around the affected vessels.

Nevertheless, secondary complications were seen on OCT-A in fourteen eyes (74%), including some findings not clearly shown on FA. Most of these were within the macular area. OCT-A was particularly effective in cases of capillary dropout, increased Foveal Avascular Zone (FAZ), telangiectasias, shunts, areas of neovascularization and other vascular aberrations. In the five cases in which OCT-A was normal, we found no evidence of other lesions in the macular region on FA other than vasculitis (perivascular leakage).

**Fig. 1:**
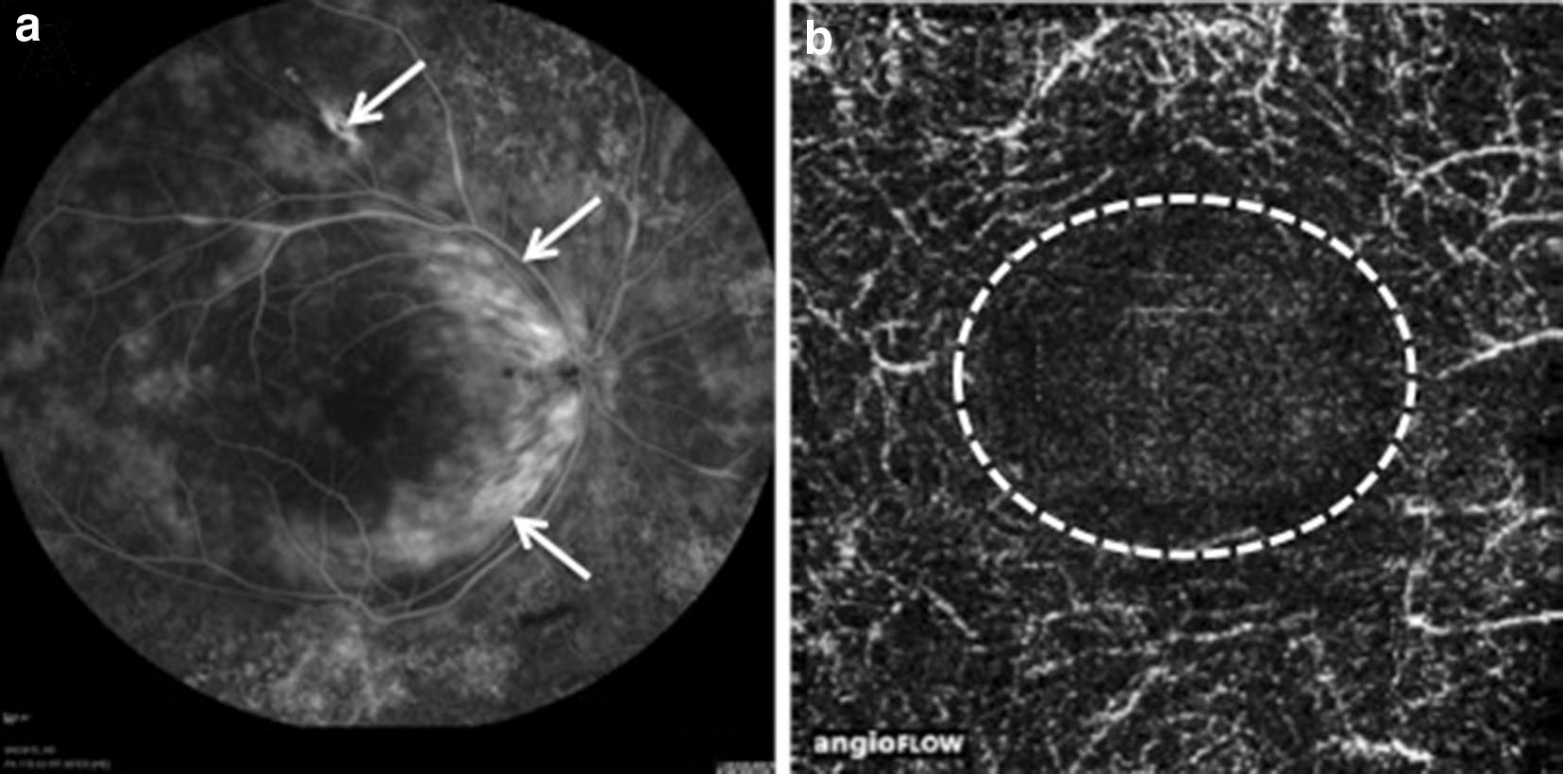
Behçet’s disease **a** FA image revealing vascular leakage in the upper temporal arcade and an extensive area of leakage (arrows) at the nasal macula, denoting active vasculitis. **b** 3 × 3 mm OCT-A scan revealing central macular deep capillary dropout with increased FAZ, about 2.4 mm^2^ of area (circle)

OCT-A facilitated the detection of vascular nonperfusion areas with capillary dropout in eight eyes (42%). In two of these (10%), there was increased FAZ (Fig. 1).

**Fig. 2:**
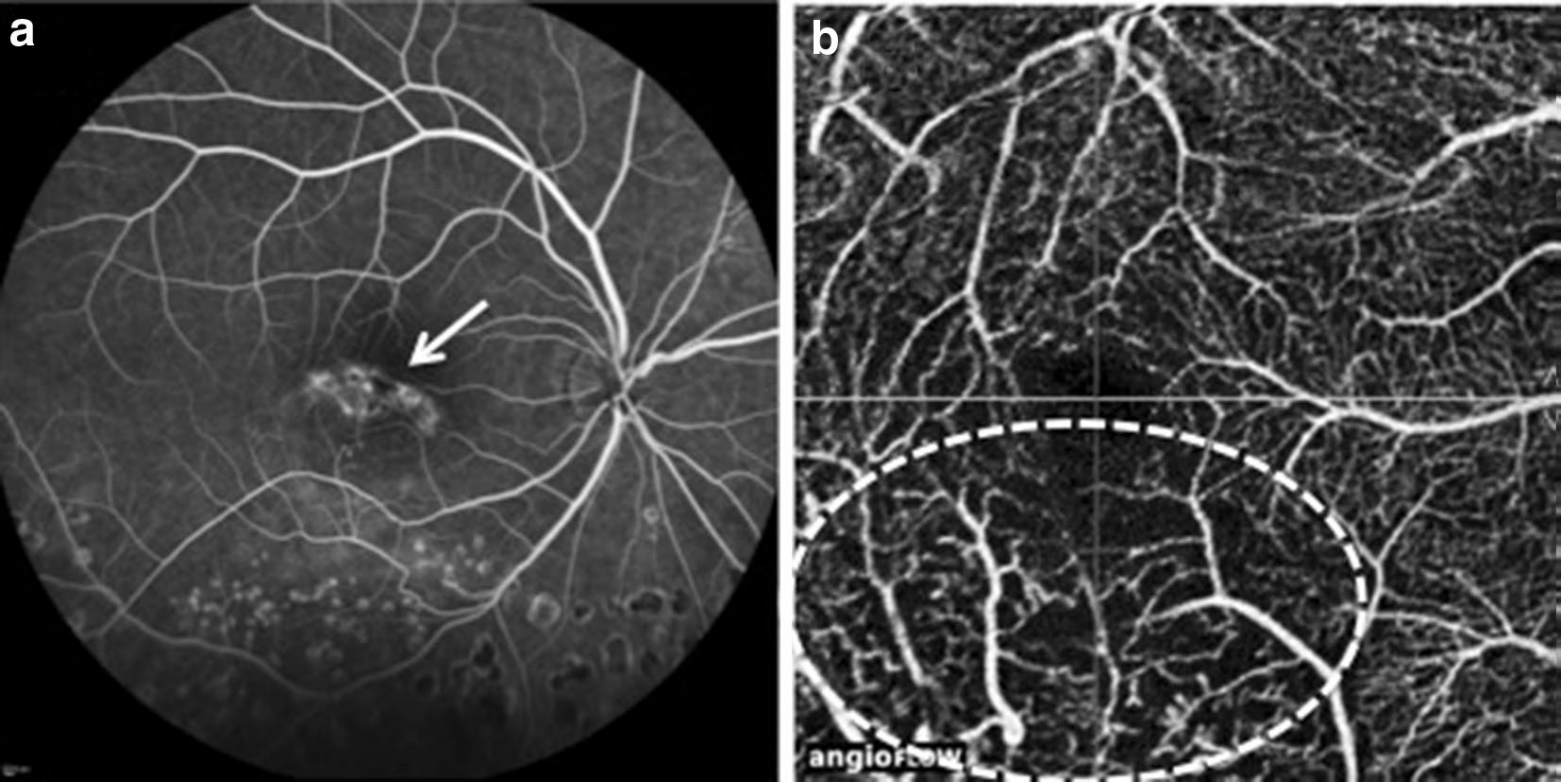
Sarcoidosis **a** FA image revealing leakage at central macula (arrow), denoting unspecific vascular abnormalities. **b** 3 × 3 mm OCT-A scan revealing temporal and inferior capillary dropout and areas of telangiectasias at lower macula (circle)

**Fig. 3:**
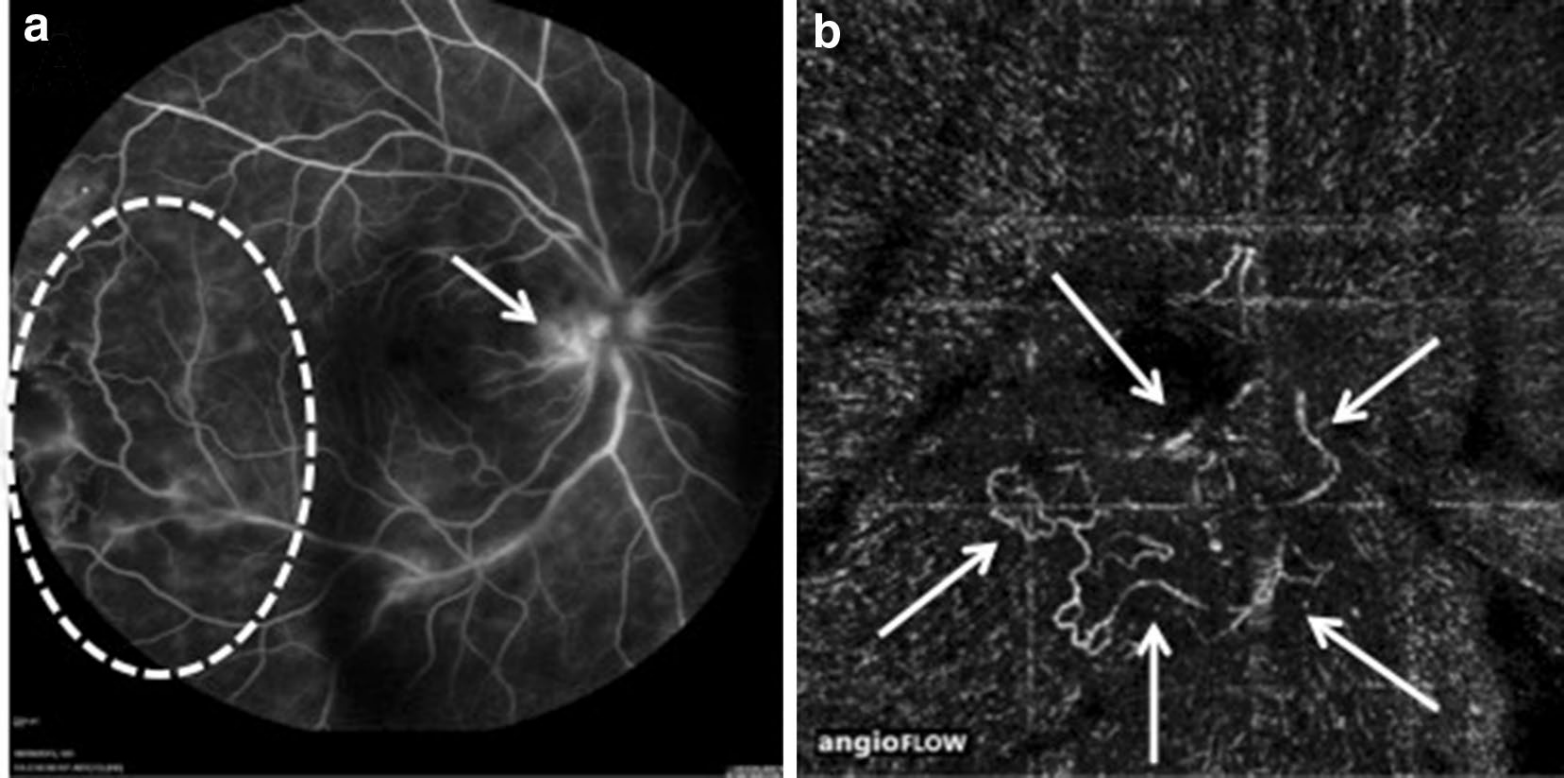
Takayasu arteritis **a** FA evidences temporal area of vascular nonperfusion associated with adjacent telangiectasias (circle) and unspecific hyperfluorescence in the peripapillary area (arrow). **b** 3 × 3 mm OCT-A scan of the optic disc shows lower peripapillary neovascular proliferation (arrows)

OCT-A revealed vascular abnormalities, including shunts and telangiectasias in four eyes (21%) (Fig. 2) and the development of neovascularization in two eyes (10%) (Fig. 3).

## Discussion

In this case series, we reported outcomes observed in 19 eyes with various forms of retinal vasculitis, either active or in remission. The OCT-A findings were compared to the FA findings in order to measure their roles in diagnosis of active retinal vasculitis as well as the presence of secondary complications.

Interestingly, in those cases where FA showed clear signs of retinal vasculitis activity, with characteristic findings of perivascular contrast leakage, OCT-A was not able to clearly detect such vascular abnormalities. The fact that OCT-A does not use contrast, combined with the restricted field of the macular zone, likely limits the detection of peripheral vasculitis by OCT-A. In addition, vitreous opacities caused by vitritis or hemorrhage, and the difficulty of central fixation in some patients with significant low vision were also factors that negatively influenced images obtained by OCT-A. OCT-A requires that the patient fixate precisely for a few seconds, whereas a usable fluorescein angiographic frame can be obtained in a fraction of a second [9].

Nevertheless, OCT-A plays a critical role in the detection of secondary complications of vasculitis that are commonly not detected by FA, including new vessels obscured by retinal hemorrhage, early peripapillary neovascular proliferation, and telangiectasias. Furthermore, the most relevant and repeated finding observed on the OCT-A exam was macular capillary dropout. In six of our cases, ischemic findings were better demonstrated by OCT-A than by FA, including two cases with increased FAZ (Table 2). In one case of Takayasu arteritis, FA was superior for identification of a peripheral nonperfusion area. Vascular abnormalities were well demonstrated by both exams, however, neovascular formations were revealed only on OCT-A. This was because confounding factors including fluorescein blood blockage and inflammatory vascular leakage did not allow FA to clearly identify these important findings (Table 2).

**Table 2:** Fluorescein angiography (FA) and optical coherence tomography-angiography (OCT-A) findings

	Only FA detected	Only OCT-A detected	Both exams detected
Vascular non-perfusion areas (capillary drop-out and increased FAZ)	1	6	2
Vascular abnormalities (shunts and telangiectasias)	0	0	4
Neovascularization	0	2	0
Total	1	8	6

Our findings are in agreement with Khairallah et al. [10], who described OCT-A findings in eyes with active Behçet’s uveitis, demonstrating that OCT-A allowed better visualization and characterization of perifoveal microvascular changes than did FA. This included disruption of the perifoveal capillary arcade, areas of retinal nonperfusion, and perifoveal capillary abnormalities, including dilated or shutting vessels. A recent study [11] also reported that OCT-A provided images with great detail regarding macular status and allowed the discrimination between superficial and deep retinal vascular plexuses that could not be demonstrated by conventional fluorescein angiography.

Ishibazawa et al. [12] reported that OCT-A could be used to study the origin of microaneurysms in patients with diabetic retinopathy. Despite this improved identification, reports have demonstrated that the number of microaneurysms was significantly lower than was obtained by conventional FA. However, OCT-A also gave the added benefit of localizing exact lesion intraretinal depths [13, 14]. Corroborating our findings, recent publications showed that OCT-A clearly depicted nonperfusion in diabetic retinopathy [12, 15]. This provided an objective automated study of macular capillary nonperfusion as a potential sign of central ischemia [16].

## Conclusion

FA continues to be an essential complementary exam to detect retinal vasculitis activity. Nevertheless, many severe secondary complications of retinal vasculitis are not well evaluated only by FA. OCT-A extends FA findings and allows complementary assessment of the stage of retinal vasculitis. As with any new technology, further studies are needed to determinate the role of OCT-A in retinal vasculitis and other retinal abnormalities. Perhaps in the future OCT-A may replace FA and may become the only imaging exam needed to detect all retinal vascular changes. However, based on our findings, we continue to recommend OCT-A assessment in addition to FA for evaluation of retinal vasculitis.

## Abbreviations

OCT-A: optical coherence tomography angiography
FA: fluorescein angiography

## References

[1] Ku JH, Ali A, Suhler EB, Choi D, Rosenbaum JT (2012). Characteristics and visual outcome of patients with retinal vasculitis. Arch Ophthalmol.

[2] Herbort CP, Cimino L, Abu El Asrar AM (2005). Ocular vasculitis: a multidisciplinary approach. Curr Opin Rheumatol.

[3] Hughes EH, Dick AD (2003). The pathology and pathogenesis of retinal vasculitis. Neuropathol Appl Neurobiol.

[4] Walton RC, Ashmore ED (2003). Retinal vasculitis. Curr Opin Ophthalmol.

[5] Nagiel A, Sadda SR, Sarraf D (2015). A promising future for optical coherence tomography angiography. JAMA Ophthalmol.

[6] Hong BK, Nazari Khanamiri H, Rao NA (2013). Role of ultra-widefield fluorescein angiography in the management of uveitis. Can J Ophthalmol.

[7] Mastropasqua R, Di Antonio L, Di Staso S, Agnifili L, Di Gregorio A, Ciancaglini M (2015). Optical coherence tomography angiography in retinal vascular diseases and choroidal neovascularization. J Ophthalmol.

[8] Ghassemi R, Brown R, Banwell B, Narayanan S, Arnold DL, Canadian G (2015). Pediatric demyelinating disease study, quantitative measurement of tissue damage and recovery within new T2w lesions in pediatric- and adult-onset multiple sclerosis. Mult Scler.

[9] Spaide RF, Klancnik JM, Cooney MJ (2015). Retinal vascular layers imaged by fluorescein angiography and optical coherence tomography angiography. JAMA Ophthalmol.

[10] Khairallah M, Abroug N, Khochtali S, Mahmoud A, Jelliti B, Coscas G (2017). Optical coherence tomography angiography in patients with behcet uveitis. Retina.

[11] de Barros Garcia JMB, Isaac DLC, Avila M (2017). Diabetic retinopathy and OCT angiography: clinical findings and future perspectives. Int J Retina Vitreous.

[12] Ishibazawa A, Nagaoka T, Takahashi A, Omae T, Tani T, Sogawa K (2015). Optical coherence tomography angiography in diabetic retinopathy: a prospective pilot study. Am J Ophthalmol.

[13] Couturier A, Mane V, Bonnin S, Erginay A, Massin P, Gaudric A (2015). Capillary plexus anomalies in diabetic retinopathy on optical coherence tomography angiography. Retina.

[14] Salz DA, de Carlo TE, Adhi M, Moult E, Choi W, Baumal CR (2016). Select features of diabetic retinopathy on swept-source optical coherence tomographic angiography compared with fluorescein angiography and normal eyes. JAMA Ophthalmol.

[15] Hwang TS, Jia Y, Gao SS, Bailey ST, Lauer AK, Flaxel CJ (2015). Optical coherence tomography angiography features of diabetic retinopathy. Retina.

[16] Hwang TS, Gao SS, Liu L, Lauer AK, Bailey ST, Flaxel CJ (2016). Automated quantification of capillary nonperfusion using optical coherence tomography angiography in diabetic retinopathy. JAMA Ophthalmol.

